# Mapping of UV-C dose and SARS-CoV-2 viral inactivation across N95 respirators during decontamination

**DOI:** 10.1038/s41598-021-98121-6

**Published:** 2021-10-13

**Authors:** Alisha Geldert, Alison Su, Allison W. Roberts, Guillaume Golovkine, Samantha M. Grist, Sarah A. Stanley, Amy E. Herr

**Affiliations:** 1 The UC Berkeley – UCSF Graduate Program in Bioengineering, University of California Berkeley, 308B Stanley Hall, Mailcode 1762, Berkeley, CA 94720 USA; 2 Department of Molecular and Cell Biology, University of California Berkeley, Berkeley, CA 94720 USA; 3 Department of Bioengineering, University of California Berkeley, Berkeley, CA 94720 USA; 4 School of Public Health, University of California Berkeley, Berkeley, CA 94720 USA

**Keywords:** Occupational health, SARS-CoV-2, Optical metrology

## Abstract

During public health crises like the COVID-19 pandemic, ultraviolet-C (UV-C) decontamination of N95 respirators for emergency reuse has been implemented to mitigate shortages. Pathogen photoinactivation efficacy depends critically on UV-C dose, which is distance- and angle-dependent and thus varies substantially across N95 surfaces within a decontamination system. Due to nonuniform and system-dependent UV-C dose distributions, characterizing UV-C dose and resulting pathogen inactivation with sufficient spatial resolution on-N95 is key to designing and validating UV-C decontamination protocols. However, robust quantification of UV-C dose across N95 facepieces presents challenges, as few UV-C measurement tools have sufficient (1) small, flexible form factor, and (2) angular response. To address this gap, we combine optical modeling and quantitative photochromic indicator (PCI) dosimetry with viral inactivation assays to generate high-resolution maps of “on-N95” UV-C dose and concomitant SARS-CoV-2 viral inactivation across N95 facepieces within a commercial decontamination chamber. Using modeling to rapidly identify on-N95 locations of interest, in-situ measurements report a 17.4 ± 5.0-fold dose difference across N95 facepieces in the chamber, yielding 2.9 ± 0.2-log variation in SARS-CoV-2 inactivation. UV-C dose at several on-N95 locations was lower than the lowest-dose locations on the chamber floor, highlighting the importance of on-N95 dose validation. Overall, we integrate optical simulation with in-situ PCI dosimetry to relate UV-C dose and viral inactivation at specific on-N95 locations, establishing a versatile approach to characterize UV-C photoinactivation of pathogens contaminating complex substrates such as N95s.

## Introduction

In response to global shortages of N95 respirators during the COVID-19 pandemic, a broad range of crisis-capacity strategies for decontamination and reuse of these complex, multilayered, made-for-single-use protective textiles have been proposed and investigated in a growing body of literature^1–6^. With established applications in water^7,8^, air^9–11^, and non-porous surface^12,13^ disinfection, ultraviolet-C (UV-C) germicidal (200–280 nm) irradiation was identified as one of the most promising and accessible methods for N95 decontamination^14,15^. UV-C decontamination of N95s has been reported for multiple types of pathogens^5,16–21^, including SARS-CoV-2 (the virus that causes COVID-19)^6,22^. Upon sufficient absorption by nucleic acids, UV-C inactivates pathogens by damaging their genetic material^12^; thus, UV-C decontamination efficacy is critically dependent on total received dose (integrated irradiance over time). While the level of decontamination necessary for N95 reuse may depend on institutional or federal guidelines, ≥ 3-log_10_ (≥ 99.9%) viral inactivation has previously been estimated to be a suitable decontamination threshold based on typical on-N95 viral load^18,23^ (“log_10_” subsequently referred to as “log”).

N95s present distinct challenges for UV-C decontamination: due to limited UV-C penetration through N95 material layers^5,21^, the applied surface dose required to decontaminate all N95 layers is orders of magnitude higher than the dose required on non-porous surfaces^21,24^, and varies between N95 models^16,18^ due to substantial differences in material composition. UV-C decontamination protocols must ensure that all N95 surfaces receive sufficient dose for pathogen inactivation, while also not exceeding the exposure threshold for material degradation, as high cumulative doses of UV-C degrade N95 material^25^. Additionally, UV-C dose is nonuniformly distributed across the complex, 3D N95 surface due to Lambert’s cosine law^26^ and self-shadowing^3^. Thus, UV-C distribution across N95 surfaces is highly dependent on N95 morphology, as well as the decontamination system and N95 positioning. Accurate UV-C dose measurements are critical to understanding pathogen photoinactivation for a variety of decontamination applications^27,28^. However, the unique considerations involved in N95 decontamination complicate determination of the UV-C dose applied to all N95 surfaces in a decontamination system, impacting both research and implementation^29,30^.

Effective implementation requires translation of robust research studies linking on-N95 surface dose to viral inactivation for a given UV-C source emission spectrum and N95 model. However, coincident on-N95 dose and viral inactivation measurements are infeasible as the measurement sensor would shadow the pathogen. Furthermore, most UV-C dosimetry tools lack sufficient spatial resolution, throughput, and angular response for on-N95 measurements. As a result, UV-C dose for N95 decontamination is typically characterized indirectly. For example, to circumvent challenges associated with making UV-C dose measurements on non-planar surfaces, many studies, including a recent study of SARS-CoV-2^6^, assess UV-C dose and viral inactivation on flat coupons of N95 material. N95 coupon studies determine the UV-C dose required for viral inactivation throughout the porous N95 material layers, but fail to capture the impact of the 3D facepiece shape on the received UV-C dose across the N95 surface. Other approaches use optical modeling to estimate the UV-C distribution across N95 surfaces from the UV-C dose measured in a single location, in order to relate approximate UV-C dose to SARS-CoV-2 inactivation^22^. Optical modeling is an attractive approach to study UV-C distribution, as it can recapitulate nearly any UV-C system to provide a high-resolution map of irradiance distribution^31–33^ via entirely user-defined system parameters. However, optical models alone cannot capture non-idealities such as irradiance fluctuations, bulb-to-bulb differences in power output, and environmental and material changes over time^12,29,34,35^. Additionally, while the modularity of optical modeling is advantageous for broad applicability, the model accuracy depends on both the optics expertise of the user and the accuracy of user-defined parameters such as the reflective and scattering properties of all materials. Thus, the high resolution and rapid iteration capabilities of optical simulations would be most valuable when coupled with in-situ validation measurements.

To this end, a promising in-situ method has recently been developed to quantify on-N95 dose using UV-C photochromic indicators (PCIs)^29^. PCIs complement simulation results by providing absolute dose measurements and empirical validation. Planar, paper-like dosimeters similar to PCIs have been shown to have ideal angular detection response^36^, though the angular response of UV-C PCIs has not been quantified. The low cost and small, flexible form factor of PCIs supports quantitative, spatially resolved and high-throughput on-N95 PCI dosimetry in the same exposure and in nearly the same on-N95 location as inoculated pathogens, minimizing confounding factors such as temporal or spatial variation^29^ and angular dependence of UV-C irradiance^26^. Thus, PCIs may comprise a cornerstone to better inform safe and effective UV-C decontamination, especially when corroborated by further study to confirm PCI angular response and suitability for readout by diverse, lower-cost color readers.

Here, to investigate the impact of UV-C dose variation on SARS-CoV-2 inactivation on N95s, we introduce a method to simultaneously map UV-C dose and SARS-CoV-2 viral inactivation across N95 respirator facepieces. We integrate two approaches for high-spatial-resolution on-N95 dosimetry: PCI quantification and optical modeling. We develop an optical modeling workflow to characterize UV-C dose distribution across N95s within a decontamination chamber to rapidly iterate on experimental design, and simultaneously inform and validate this model using in-situ PCI dose quantification. From the high-resolution simulated N95 dose maps, we identify pairs of proximal measurement sites receiving equivalent UV-C dose in order to measure UV-C dose at SARS-CoV-2 inoculation sites within the same UV-C exposure. For the first time, we apply quantitative in-situ PCI dosimetry to simultaneously quantify UV-C dose and SARS-CoV-2 inactivation across a model N95 facepiece (intra-N95) at multiple locations (intra-chamber), providing new, practical insight into how N95 facepiece shape impacts decontamination efficacy.

## Materials and methods

### Inter-UV-C chamber and radiometer assessment

All UV-C decontamination experiments were performed with Spectronics XL-1000 UV-C chambers with BLE-8T254 low-pressure amalgam bulbs. Irradiance was measured using calibrated, NIST-traceable ILT 1254/TD UV-C radiometers (International Light Technologies, ILT) and corresponding ILT DataLight III meter software. A custom notch in the UV-C chamber doors allowed a cable to pass through for in-situ radiometer measurements. One chamber and radiometer were used exclusively in a biosafety level 3 (BSL-3) laboratory for SARS-CoV-2 inactivation experiments, while another set was used for all experiments outside BSL-3. The UV-C irradiance over time and space within the two chambers were concordant (Supplementary Fig. S1), as were measurements from the two radiometers (Supplementary Fig. S2).

### PCI measurements

For UV-C dose measurements, PCIs (UVC 100 Dosimeter dots, American Ultraviolet; 25.4 mm diameter) were cut into quarters prior to use. D65/10° L*a*b* PCI color was measured using an RM200QC spectrocolorimeter (X-Rite, large aperture setting) and/or Color Muse colorimeter (Variable, Inc, with Variable color app). The Color Muse was aligned over the PCI using a template (Supplementary Fig. S3). PCI quantification was performed as described previously^29^, summarized in Note S1. The PCI dynamic range was defined as the dose range over which relative uncertainty (defined as half the width of the 95% confidence interval on UV-C dose measurements, divided by measured dose) was < 10%. To measure doses beyond the PCI dynamic range on planar surfaces, a 1.1 mm-thick Borofloat glass attenuator (25.4 mm width and length, 80/50 scratch/dig quality, Precision Glass & Optics 0025-0025-0011-GE-CA) with 12.4% ± 0.4% UV-C transmittance (measured in a UV-C chamber) was placed over the PCI. We generated calibration curves specific to the PCI batch, attenuator, and colorimeter to quantify UV-C dose from PCI color change (CIEDE2000 ΔE) with respect to an unexposed reference (Supplementary Figs. S4, S5). PCI angular response was characterized by measuring the RM200QC-measured dose quantified from PCIs exposed to a UV-C point-like source at different angles of incidence (Supplementary Fig. S6).

For all ratios of two PCI measurements (e.g., fold difference in on-N95 dose), other than cases where PCI measurements are normalized to a maximum PCI reading, we report total error: the root sum square of standard deviations associated with both replicate variation and propagated uncertainty in PCI dose estimation. All other error values report the standard deviation of replicate measurements.

### Optical model

To create a model of the respirator compatible with the optical modeling software, a 3M 1860 N95 with straps removed was scanned using a Creaform Go!SCAN 3D. After additional pre-processing, the scan of the N95 was positioned within a CAD model of the UV-C chamber (Supplementary Fig. S7). The entire assembly was then imported into non-sequential mode in Zemax OpticStudio Pro (Version: 20.3) and exploded into individual parts. Parts not essential to the optical model (e.g., screws, hinges, etc.) were ignored during simulations. UV-C source and surface parameters are listed in Table S1. The N95 CAD object was converted to an absorbing detector, consistent with a previous study that approximated on-N95 UV-C distribution using an absorbing spherical detector^31^, and positioned and/or duplicated to match in-situ chamber locations. All simulations were performed with “Use Polarization”, “Scatter NSC Rays”, “Split NSC Rays” and “Ignore Errors” engaged. Detector data were exported and analyzed using custom MATLAB scripts. Because the optical model may not accurately predict absolute dose due to environmental fluctuations, simulation results were normalized to the maximum value within the analyzed domain (e.g., entire chamber and/or N95(s)).

### UV-C dose distribution on chamber floor

UV-C dose distribution across the chamber floor was characterized in situ at 15 evenly spaced locations (Supplementary Fig. S8) using PCIs as described previously^29^; briefly, all 15 PCIs were simultaneously exposed to ~ 100 mJ/cm^2^, then read with the RM200QC within 600 s. Peak UV-C irradiance within a 15 s exposure was also measured at 14 of these locations sequentially using a radiometer (the built-in chamber sensor obstructed placement at one location). Simulated UV-C dose at each location was extracted from the optical model using custom MATLAB scripts.

### UV-C dose distribution across N95 facepieces

#### In-situ measurements

To empirically measure on-N95 UV-C dose, PCIs with backing removed were adhered to the N95 facepiece, exposed, and subsequently removed for color quantification. To facilitate comparison to simulation, each PCI location on the N95 was recorded by measuring the PCI: (1) corner height (*C*), (2) highest point height if not corner height (lowest point height if corner is highest) (*h*), (3) rotation along the N95 surface (Φ, Supplementary Fig. S9), and (4) lateral distance from either the nosepiece-to-chin midline or side-to-side seam. N95 straps were removed to minimize shadowing and variability in N95 tilt. A printed floor map ensured reproducible N95 positioning in the chamber, with nosepieces toward the door (Supplementary Fig. S10).

#### Optical model

To characterize on-N95 UV-C dose from simulations, average values at specific in-situ PCI locations were extracted from the N95 detector simulation data using a custom MATLAB script. Briefly, N95 detector data were imported into MATLAB. The outline of each PCI was plotted on top of the simulated N95 dose map using the spatial parameters described above. The vertical height of the PCI (*d*) was then defined as |*C-h*|. The angle of rotation toward the N95 surface (α, Supplementary Fig. S9) of the PCI was calculated based on geometry. When the corner is either the highest or lowest part of the PCI, α is defined as Eq. (1):

1 $$\alpha ={cos}^{-1}\frac{d}{r}$$

where *r* is the radius of the PCI.

When the corner is not the highest or lowest point of the PCI, α is defined as Eq. (2):

2 $$\alpha ={cos}^{-1}\frac{d}{r*sin(\Phi )}$$

where Φ is defined as the angle between the horizontal axis and highest PCI point.

The average on-N95 value from simulation was calculated as the mean value of the data points contained within the PCI perimeter, determined using the “inpolygon” function on a 2D projection of N95 data points and PCI outlines (Supplementary Fig. S9).

### Heatmap plots

All heatmaps of UV-C dose or irradiance were generated with the ‘inferno’ perceptually uniform, colorblind-friendly colormap, which was created by Stéfan van der Walt and Nathaniel Smith and adapted from Python’s matplotlib for use in MATLAB by Ander Biguri^37^.

### SARS-CoV-2 preparation and handling

For all viral inactivation experiments, SARS-CoV-2 preparation, stock titration, inoculation, and TCID_50_ assay were performed as described in Note S2. In brief, 3 aliquots of 16.67 μL (50 μL in total) of SARS-CoV-2 stock at 8 × 10^7^ TCID_50_/mL were loaded per N95 inoculation site and dried for 3.5 h at room temperature in a biosafety cabinet. After UV-C exposure, inoculation sites were excised with a 12 mm-diameter biopsy punch (MedexSupply ACD-P1250), incubated in DMEM supplemented with 10% fetal bovine serum, 100 U/mL penicillin, and 100 μg/mL streptomycin for ≥ 30 min, and viable SARS-CoV-2 virus was quantified by a TCID_50_ assay. All study procedures were approved by the UC Berkeley Committee for Laboratory and Environmental Biosafety and conducted in agreement with BSL-3 requirements.

### SARS-CoV-2 dose response on N95 coupons

The UV-C dose response of SARS-CoV-2 was assessed by measuring viral inactivation on 3M 1860 N95 coupons inoculated with SARS-CoV-2 and exposed to different UV-C doses. By mapping UV-C irradiance across the chamber floor, we identified 5 locations of equivalent irradiance at which to place a radiometer, PCI, and 3 inoculated N95 coupons (Supplementary Figs. S11, S12). PCIs and coupons were placed on custom-built platforms to match the height of the radiometer sensor. Platforms were built from laser-cut (HL40-5G-110, Full Spectrum Laser) pieces of 3.175 mm-thick acrylic (McMaster Carr 85635K421), joined with epoxy (J-B Weld 50176). Printed maps on the chamber floor and platforms ensured consistent positioning from run-to-run (Supplementary Fig. S13).

For SARS-CoV-2 inactivation experiments, 3 replicate inoculated coupons were simultaneously irradiated with a given UV-C dose. Coupons (15 mm × 20 mm) were cut from the edge of N95s to include the raised, sealed seam to minimize layer separation. The seam did not prevent the coupons from lying flat during UV-C exposure. Both a radiometer and PCI (with Borofloat attenuator for doses beyond the PCI upper limit of quantification) were used to quantify in-situ UV-C dose applied during each exposure. Exposure time was estimated by dividing the target dose by the irradiance at the coupon platform (~ 6.4 mW/cm^2^ after bulb warm-up). To account for output degradation^29^ (Supplementary Fig. S1), exposure time was optimized by comparing the dose measured by the radiometer during a test exposure to the target dose.

To evaluate whether inactivation is affected by any temperature increase in the chamber during UV-C exposure, SARS-CoV-2 survival on unexposed coupons kept either at room temperature or within the UV-C chamber but shielded from UV-C during irradiation (i.e., ‘heating control’ coupons) was compared. The inoculated heating control coupon was placed on a platform of the same height as the coupon and PCI platforms, and in a location receiving approximately the same irradiance. A custom acrylic cover, verified to block all UV-C, was then placed on top of the heating control coupon, so that the coupon would be exposed to the temperature rise in the chamber but not to UV-C. A PCI was placed near the heating control coupon, under the acrylic cover, to verify that the heating control coupon is not irradiated with UV-C.

After each exposure, PCI(s) were measured with both the RM200QC and the Color Muse. Biopsies were excised from all irradiated coupons, as well as one unexposed control coupon stored at room temperature outside the UV-C chamber. A TCID_50_ assay was performed to assess SARS-CoV-2 viability (Note S2).

### Paired measurements of UV-C dose and SARS-CoV-2 inactivation on N95s

To simultaneously measure UV-C dose and SARS-CoV-2 inactivation on 3M 1860 N95 facepieces, PCIs (without attenuator) were affixed to N95s at each chosen dose measurement site, and accompanying SARS-CoV-2 inoculation sites outlined in advance to facilitate accurate viral deposition. SARS-CoV-2 was inoculated at each paired inoculation site. After drying, two N95s (‘corner’ and ‘front’ N95s) were placed in a UV-C chamber after bulb warm-up. To monitor dose during each exposure, a radiometer and PCI were also placed at their respective positions near the two corners of the chamber floor (Supplementary Fig. S10). After a 10 s UV-C exposure, PCIs were removed from the N95s and measured with both the RM200QC and the Color Muse. SARS-CoV-2 inoculation sites as well as an unexposed room temperature control coupon were excised following each UV-C exposure. The correlation coefficient and significance of correlation between UV-C dose and SARS-CoV-2 inactivation was determined with the *corrcoef* function in MATLAB. The linear regression and confidence intervals on the linear fit parameters were determined with the MATLAB functions *fit* (with the ‘poly1’ model) and *confint*, respectively. The 95% prediction intervals on the linear regression function were determined using the MATLAB function *predint* with simultaneous functional bounds.

For all experiments (including the scan used for optical simulation, in-situ UV-C dose distribution measurements, and SARS-CoV-2 inactivation measurements), expired 3M 1860 N95s were used to preserve non-expired N95s for healthcare workers.

## Results and discussion

### Measuring UV-C dose and SARS-CoV-2 inactivation on and across N95s

**Figure 1:**
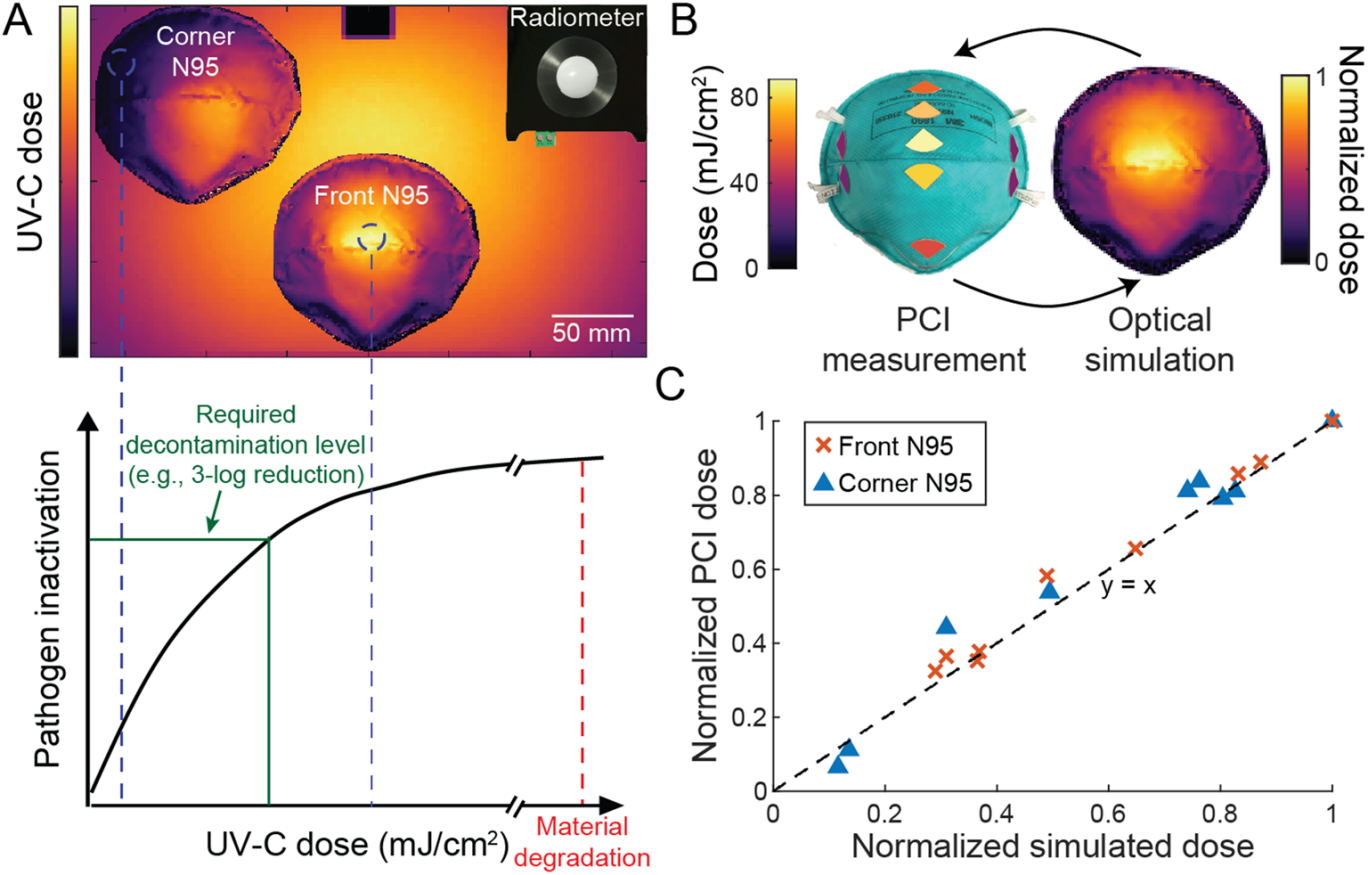
Integrated optical modeling and in-situ PCI measurement pipeline for simultaneous and near-coincident on-N95 UV-C dose and viral inactivation measurements. (**A**) Schematic highlighting how UV-C dose received across complex N95 surfaces can vary substantially, creating a narrow range of UV-C doses that deliver sufficient dose for pathogen inactivation while not exceeding the exposure threshold for material degradation. (**B**) In-situ PCI measurements and optical simulation results were used in tandem to inform and rapidly iterate on experimental design. (**C**) Comparison of normalized in-situ PCI and simulated doses at seven discrete locations on N95s in two different chamber positions. To assess agreement between PCI dose measurements and normalized simulation data (both characterized with one N95 in the chamber at a time), the datasets for each N95 position were normalized to the maximum observed dose for the individual N95. Black line (y = x) indicates where the data would lie if PCI and simulation measurements were equal.

In this study, we sought to understand the impact of N95 shape and placement on SARS-CoV-2 inactivation and how variation in inactivation relates to UV-C dose received across the N95 surfaces (Fig. 1A). Building upon previous work quantitatively relating PCI color change to received UV-C dose^29^, we introduce simultaneous measurement of SARS-CoV-2 inactivation and UV-C dose on N95 facepieces. We extend characterization of the quantitative PCI dosimetry method^29^ in two ways. First, we measured the angular response of PCIs and verified a near-ideal response (Supplementary Fig. S6), confirming that PCIs are suitable to measure UV-C dose on non-planar surfaces. Second, to increase accessibility of the PCI dosimetry method, we compared the performance of a substantially lower-cost colorimeter to the previously reported spectrocolorimeter (Supplementary Fig. S4). Applying this PCI dosimetry method, we paired PCI UV-C dose measurements with SARS-CoV-2 inactivation measurements to characterize the received dose and resulting viral inactivation variation across N95 facepiece surfaces.

Towards the study goal of assessing impact of N95 shape and placement on received UV-C dose and SARS-CoV-2 inactivation, we first identified the dynamic range of the UV-C dose–response curve. Only doses within this range will elucidate the variable relationship between UV-C dose and viral inactivation. The physical setup and exposure time of N95s in a decontamination chamber and the SARS-CoV-2 inoculation sites were then optimized to receive doses spanning that dynamic range. To perform this non-trivial optimization, we implemented both in-silico optical ray-trace modeling and in-situ experimental PCI quantification. We iterated between high-resolution modeling predictions and the more accurate on-N95 PCI-based UV-C dose measurements (Fig. 1B).

### Building and validating optical model of a UV-C decontamination system

After optically modeling the UV-C decontamination chamber, we observed that normalized simulation dose measurements differ from normalized in-situ radiometer measurements by an average of 4.7% ± 4.5% at 14 unique locations across the chamber floor (Supplementary Fig. S11). Assuming spatially invariant fluctuations, the normalized irradiance and normalized dose distribution within the system are equal. Therefore, the terms “normalized irradiance” and “normalized dose” are used interchangeably to compare to in-situ results, depending on the in-situ measurement approach (i.e., radiometer or PCI). From a 3D scan of a 3M 1860 N95 imported into the virtual UV-C chamber, normalized simulation measurements differ from normalized in-situ PCI measurements by an average of 6.0 ± 6.2% across the facepiece centrally positioned near the chamber door (‘front N95’) (Fig. 1C). The largest discrepancy on the door-facing N95 surface saw simulation underestimate the normalized PCI dose by ~ 16%. For an N95 positioned in the chamber rear corner (‘corner N95’), simulation differed from in-situ PCI measurements by an average of 18 ± 25%, with the largest discrepancies again occurring on the wall-facing N95 surfaces (Supplementary Fig. S14). Differences between the simulation and in-situ measurements may arise due to N95-to-N95 shape variability (Supplementary Fig. S15), differences between true and modeled surface properties, and higher relative uncertainty of low-dose PCI measurements. Overall, however, on-N95 dose measurements correlate with simulation measurements at corresponding locations (Fig. 1C). Thus, after validating the agreement between the simulation and in-situ measurements across both the chamber floor and an N95 in multiple chamber locations, we coupled the two measurement tools to design and optimize paired UV-C dose and SARS-CoV-2 inactivation experiments, leading to the first demonstration of simultaneous on-N95 viral inactivation and UV-C dose measurements to date.

### Establishing dose–response for SARS-CoV-2 viral inactivation by UV-C

**Figure 2:**
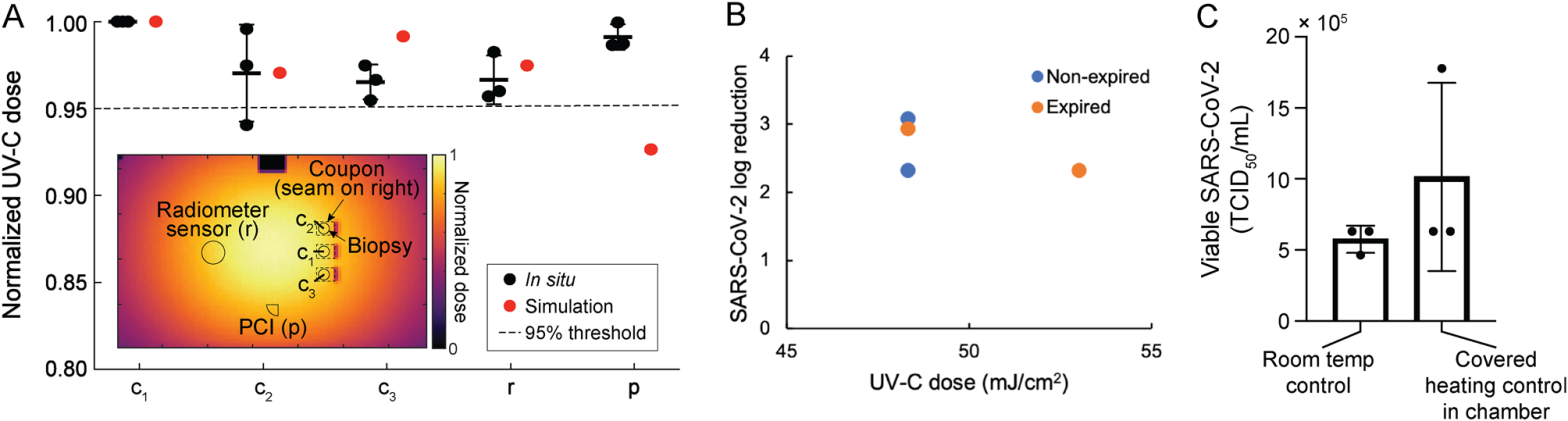
Experimental design and controls for SARS-CoV-2 dose response measurement on N95 coupons. (**A**) Using the optical model and validating in situ, five locations within the decontamination chamber receiving similar UV-C doses (< 5% variation between mean in-situ dose measurements at each location) were identified. To inform biopsy location, the optical model also assessed the impact of each coupon seam (Figure S17; modeled as 15 mm wide × 2.5 mm tall × 1 mm thick absorbing rectangular volumes at the right-hand side of each coupon) on UV-C distribution. In-situ measurements were made using PCIs to simultaneously measure dose received at the PCI and 3 coupon locations while simultaneously recording irradiance with the radiometer. Mean and standard deviation are indicated for the in-situ measurements. (**B**) SARS-CoV-2 inactivation did not depend on the expiration status of 3M 1860 N95 coupons used in this study (N = 2 replicates/condition). (**C**) Comparison of SARS-CoV-2 survival on N95 coupons at room temperature and within the chamber shielded from UV-C during illumination demonstrates that chamber heating does not impact inactivation. p > 0.9999, Wilcoxon matched-pairs signed rank 2-tailed test. Bars denote mean of N = 3 measurements per condition; whiskers denote standard deviation; points denote individual measurements.

In order to quantify the UV-C dose dependence of SARS-CoV-2 viral inactivation without the added complexity of the N95 facepiece shape, we first considered SARS-CoV-2 viral inactivation using UV-C on coupons of N95 material. Simulation and in-situ measurements identified and validated five locations in a UV-C decontamination chamber that receive equivalent UV-C irradiance (< 5% variation, Fig. 2A) for location-paired UV-C dose and SARS-CoV-2 inactivation measurements on N95 coupons. We simultaneously exposed triplicate coupons inoculated with SARS-CoV-2 while recording the applied dose using both a radiometer and a PCI (Supplementary Fig. S16). As the dynamic range of the PCIs measured with either color reader was insufficient to measure UV-C doses ⪆260 mJ/cm^2^ (Supplementary Fig. S4), for these higher doses we placed 1.1 mm-thick Borofloat glass over the PCI on the flat PCI platform to attenuate incident UV-C irradiance and extend the PCI dynamic detection range. We observed an extended upper limit of quantification (ULOQ) of 1,853.2 mJ/cm^2^ for the Borofloat-PCI pair when PCI color is measured with the spectrocolorimeter, compared to 261.4 mJ/cm^2^ without the attenuator (Supplementary Fig. S4). When using the lower-cost colorimeter, Borofloat extended the ULOQ from 168.1 mJ/cm^2^ to 802.6 mJ/cm^2^ (Supplementary Fig. S4). While the ULOQ of the Borofloat-PCI pair when using the lower-cost colorimeter was lower than some of the UV-C doses included in the SARS-CoV-2 dose–response measurements, we observed good agreement in estimated dose using both color readers to measure all PCIs in SARS-CoV-2 experiments (Supplementary Fig. S4). Borofloat was only paired with PCIs on planar surfaces (not on-N95), as Borofloat transmittance is expected to depend on incident angle, yielding non-ideal angular response.

First, we evaluated the effect of N95 expiration status on viral inactivation efficacy. By measuring SARS-CoV-2 inactivation on expired (i.e., past the manufacturer-recommended shelf life) and non-expired coupons (N = 2 coupons per condition), we observed minimal impact of expiration status on UV-C decontamination efficacy among the batches of 3M 1860 N95s tested (Fig. 2B). Thus, expired N95s were used for all experiments to preserve non-expired N95s for healthcare workers.

**Figure 3:**
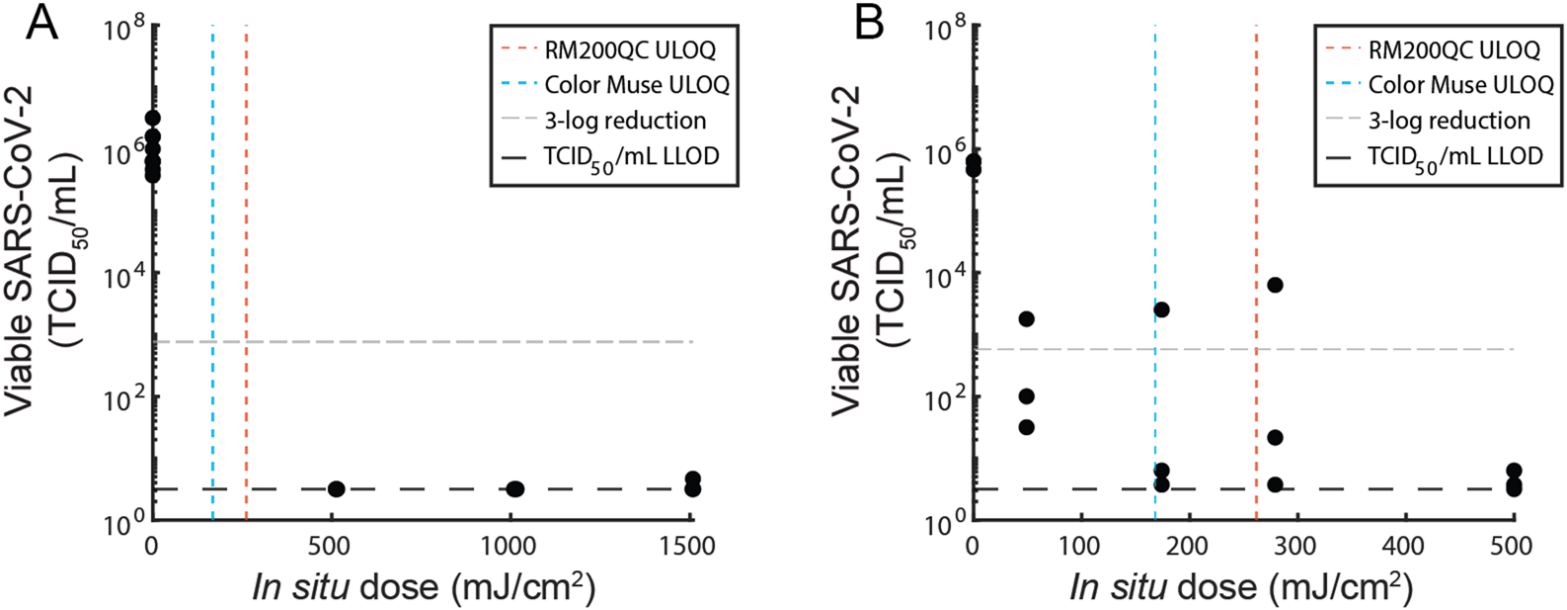
Dynamic range of SARS-CoV-2 dose response curve on N95 coupons is between 0 and 50 mJ/cm^2^. SARS-CoV-2 recovery on 3M 1860 N95 coupons as a function of in-situ UV-C dose measured using a radiometer, for (**A**) doses between 0 and 1500 mJ/cm^2^, and (**B**) doses between 0 – 500 mJ/cm^2^. ULOQ = upper limit of quantification. LLOD = lower limit of detection. N = 3 replicates per condition.

To elucidate the SARS-CoV-2 dose–response curve, we measured SARS-CoV-2 viral activity from N95 coupons after exposure to applied UV-C doses ranging from 500–1,500 mJ/cm^2^. The applied UV-C range was selected based on previous results demonstrating that ≥ 1000 mJ/cm^2^ UV-C dose is required for 3-log inactivation of SARS-CoV-2 analogs on the majority of N95 models tested^16–18^. For all replicates exposed to 500–1500 mJ/cm^2^, we observed > 5-log SARS-CoV-2 reduction on N95 coupons (Fig. 3A). Furthermore, any remaining virus was below the limit of detection of the TCID_50_ assay for all but one replicate, signifying that lower doses are required to identify the dynamic range of the dose–response curve of our assay.

We next assessed an applied UV-C dose range of 50–500 mJ/cm^2^. For these lower UV-C doses, we observed an average of > 3-log reduction of viable SARS-CoV-2 virus at all doses (Fig. 3B), with no significant differences observed between non-zero UV-C dose conditions (N = 3 replicates, Kruskal–Wallis test with Dunn’s multiple comparison test, α = 0.05, p = 0.2529). We also confirmed that any temperature increase in the chamber during exposure does not contribute to SARS-CoV-2 inactivation (Note S3, Fig. 2C). We postulate that the > 20 × higher SARS-CoV-2 UV-C susceptibility observed in this study as compared to previous literature is likely attributable to two factors. First, SARS-CoV-2 was inoculated without a soiling agent (e.g., sweat or sebum surrogates); soiling agents can decrease UV-C inactivation by 1–2 logs^16,38^. Second, the 3M 1860 N95 material was very hydrophobic (water contact angle > 90°, Supplementary Fig. S17), and deposited viral samples ‘beaded’ on the facepiece surface. Greater UV-C decontamination efficacy has generally been observed on hydrophobic N95 models^16^, which we hypothesize may be due to the greater proportion of virus inoculated on the outer N95 layers. Because the outer N95 layers receive more UV-C dose than inner layers^21^, inactivation on hydrophobic N95s may more closely resemble nonporous surface decontamination, on which lower UV-C doses (~ 4.3 mJ/cm^2^) have been shown to yield > 3-log reduction of SARS-CoV-2^39^. Droplet imbibition into porous matrices is a complex process that depends on properties of the fluid and substrate^40^, differences in inoculation volume and solution, and N95 material, all of which may influence the proportion of virus which penetrates into inner N95 layers. Thus, the system scrutinized here is an idealized model system and the SARS-CoV-2 dose response behavior observed is not anticipated to represent SARS-CoV-2 inactivation in clinical settings, where different N95 models and soiling are expected to substantially increase the necessary UV-C dose. Given the results and precision of the TCID_50_ assay (Fig. 3B), and the anticipated single- or two-stage exponential inactivation of virus with increasing dose^6,12,41^, we expect the dynamic range of our measured dose–response curve to exist between 0 and 50 mJ/cm^2^. Thus, we aimed to deliver UV-C doses within this range to map SARS-CoV-2 inactivation differences and UV-C dose nonuniformity to the complex 3D geometry of N95 facepieces (i.e., comparing among facepiece locations).

### On-N95 UV-C mapping informs design of near-coincident UV-C dose and SARS-CoV-2 inactivation measurements

Having established the UV-C dose response of SARS-CoV-2 on flat N95 coupons, we next investigated the magnitude of N95 shape-induced UV-C dose variation, as received UV-C is dependent on incident angle and distance from the UV-C source. Concomitantly, we sought to understand how the nonuniform on-N95 UV-C dose translated to SARS-CoV-2 viral inactivation efficacy. We aimed to map SARS-CoV-2 inactivation differences and UV-C dose nonuniformity across the N95 facepiece by simultaneously quantifying on-N95 dose with in-situ PCI measurements and SARS-CoV-2 inactivation via TCID_50_. The tandem approach allowed simultaneous measurement at multiple locations on intact N95 facepieces: a measurement not feasible with radiometers or viral inactivation measurements alone.

Because a PCI placed at the SARS-CoV-2 inoculation site would shadow the virus inoculum, we used optical simulation to identify pairs of adjacent on-N95 measurement sites which receive equal dose. With paired measurement sites, the UV-C dose received by a SARS-CoV-2 inoculation site can be monitored using a PCI placed at the proximal equivalent-dose site. Optical simulation rapidly reports the irradiance distribution across easily tunable N95 configurations with high spatial resolution, facilitating identification of: (1) *locations* to make on-N95 measurements that sample the range of delivered UV-C doses, and (2) *measurement sites* within each location receiving the same dose. Each location must be large enough to house two proximal measurement sites each ~ 13 mm in diameter.

**Figure 4:**
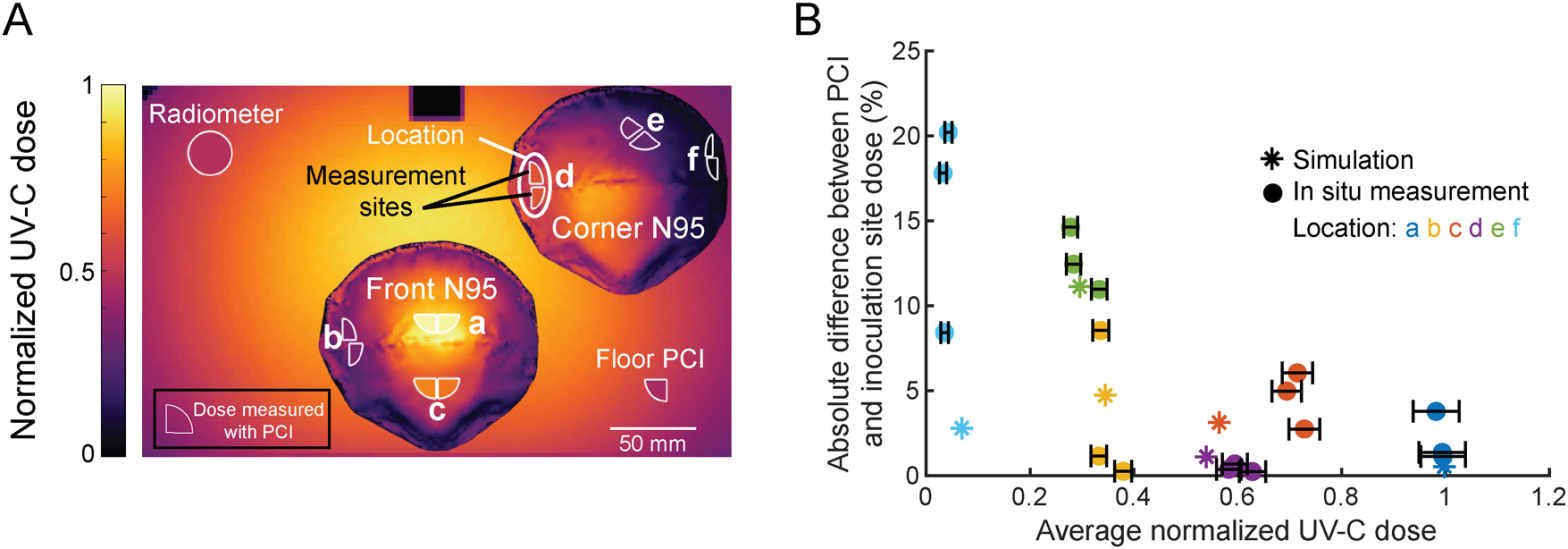
Optical simulation and PCIs identify paired measurement sites receiving equivalent UV-C dose within locations on N95 facepieces receiving a wide range of UV-C doses for paired dose and viral inactivation measurements. (**A**) Optical simulation of UV-C dose distribution over two 3M 1860 N95 facepieces in the UV-C chamber, overlaid with PCIs at paired measurement sites for viral inactivation and dose measurement. Heatmap shows simulated UV-C dose (normalized to the maximum dose in the chamber). PCI fill color represents the mean dose measured with PCIs in situ across triplicate measurements. (**B**) Comparison of dose differences within paired measurement sites. Data are colored by on-N95 location. Horizontal error bars on measured values represent the 95% confidence interval in estimated dose (Note S1).

We first used optical simulation to characterize the UV-C dose distribution across the surface of multiple N95s within the chamber. To increase decontamination system throughput, multiple N95s are often irradiated simultaneously^42,43^, but care must be taken to ensure all N95s receive sufficient dose. Additionally, N95s must be separated to prevent cross-contamination. In the studied decontamination system, three N95s can be staggered within the chamber (e.g., two in the back, one in the front). Given the lateral symmetry in dose distribution within the chamber (Supplementary Figs. S1, S11), we characterized UV-C dose distribution across two N95s in the unique positions in this ‘maximal-throughput’ layout, which we call ‘front’ and ‘corner’ (Fig. 4A). From the simulated UV-C dose map across these N95s, we identified six discrete locations (a-f in Fig. 4A) which sample the dose range. At locations a, b, d, and e, UV-C dose measured with PCIs in situ is 3.3% ± 7.6% greater than simulated dose (Fig. 4A). At location f, in-situ UV-C dose is 46.4% ± 7.6% lower than simulated dose, in line with our previous findings that simulation overestimated in-situ dose by 78% near that location (Supplementary Fig. S14). Similarly, the largest difference between simulated and in-situ UV-C dose on the ‘front' N95 is at location c, where simulated dose is 26% ± 3% lower than the in-situ dose, consistent with our previous finding that simulation underestimated the in-situ dose by 16% near the nosepiece of the front N95 (Supplementary Fig. S14). The discrepancy between simulation and in-situ UV-C dose measurements at select on-N95 locations highlights the importance of complementary in-situ measurements. In-situ measurements may be particularly valuable for other N95 models with more complicated geometry (e.g., flatfold, pleated, or duckbill), which may exhibit greater N95-to-N95 variability in shape. For example, small differences between simulated and in-situ orientation of pleats or ridges with respect to UV-C sources could lead to large differences in resulting UV-C dose distribution due to shadowing^18^ that may be difficult to fully capture with simulation measurements alone.

Within each location, the high-spatial-resolution map of simulated dose was used to identify two proposed measurement sites; dose at each site was then measured in situ using PCIs. Note that while measurement sites are often proximal to one another, the high-resolution simulation results established that some measurement sites receive the most similar dose when slightly offset (e.g., the sites at location b) due to the irregular N95 facepiece geometry and the off-center positioning of the N95s in the chamber. We observed that for locations receiving normalized UV-C doses > 0.34 (normalized to the maximum on-N95 dose), the doses across each proximal pair of PCI and inoculation sites were within 6.0% of each other, both in simulation and when measured in situ. For normalized UV-C doses ≤ 0.34 (at more steeply sloped and/or shadowed locations), the simulated doses at each pair of measurement sites were within 11.1% of each other and the doses measured in situ were within 11.8% ± 6.0% of each other (Fig. 4B). Differences between paired sites may be larger at locations with greater curvature (e.g., location e), where PCI angle (and thus, received UV-C dose) is more sensitive to run-to-run variation in PCI placement as well as N95 morphology. At locations with normalized UV-C doses ≤ 0.34, the higher relative uncertainty of PCI quantification at low doses may also contribute to a greater difference in dose at proximal sites. We quantified a relative uncertainty of ~ 20% at the lowest-dose (~ 5 mJ/cm^2^) location, compared to a relative uncertainty of ~ 5% at all other locations when PCIs are measured with the RM200QC (Supplementary Fig. S4B).

### Intra-chamber variation in UV-C dose and SARS-CoV-2 inactivation

Having identified paired on-N95 measurement sites receiving equivalent dose, simultaneous measurements of UV-C dose and SARS-CoV-2 inactivation on intact N95s could be performed. We assessed N95s placed at the front and corner positions in the decontamination chamber and chose an exposure time such that dose received across the N95 surfaces would span the dynamic range of 0–50 mJ/cm^2^ determined from the coupon study (Fig. 3B). For analysis, both UV-C dose and SARS-CoV-2 log reduction were normalized to the respective maximum value measured in the system within each replicate UV-C exposure.

**Figure 5:**
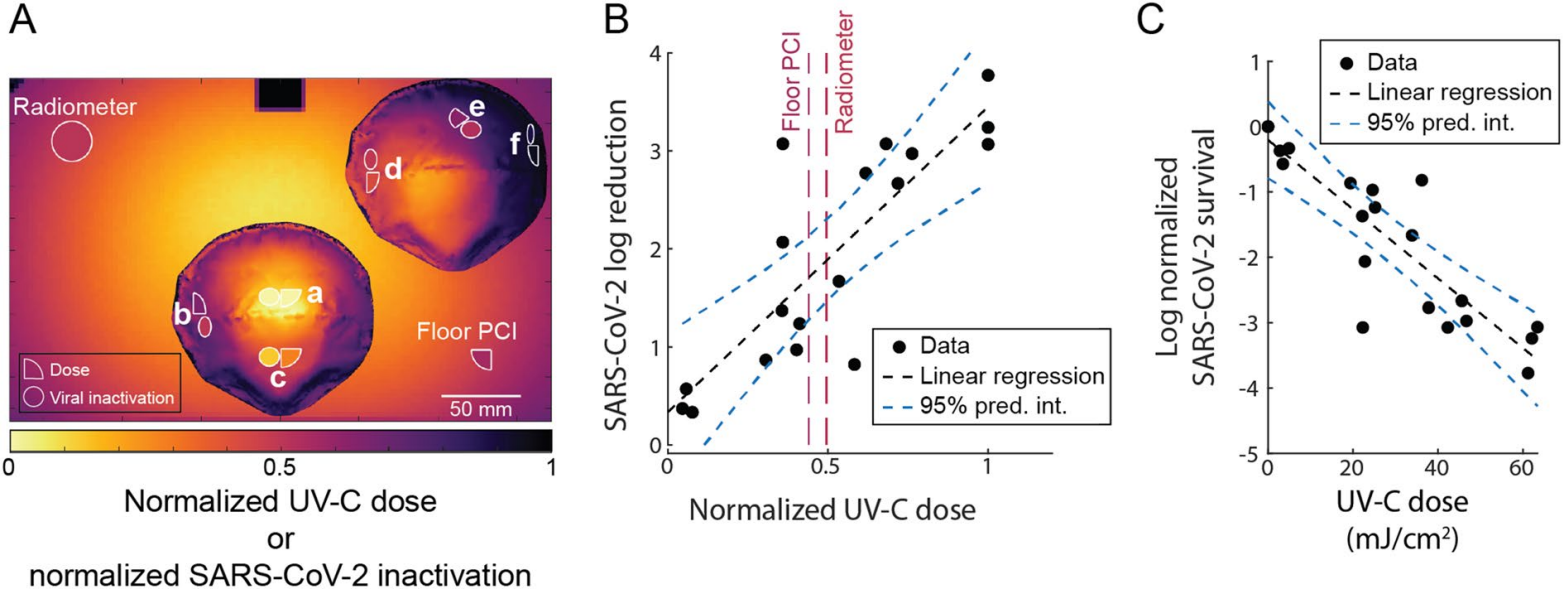
Paired on-N95 measurements of UV-C dose and SARS-CoV-2 inactivation show correlated, several-fold variation in dose and inactivation across one decontamination chamber. (**A**) Average normalized UV-C dose (quarter-circles on-N95) and SARS-CoV-2 inactivation (circles on-N95) at measured locations on front and corner N95, colored by the normalized value. UV-C doses at two corners of the chamber floor were measured by a radiometer (large circle) and PCI (quarter-circle). Values are normalized to measurements at the apex of the front N95. Surrounding heatmap shows simulated UV-C dose. (**B**) SARS-CoV-2 inactivation on N95 facepieces is proportional to UV-C dose received. Selected locations on two N95 facepieces in the Spectronics XL-1000 UV-C chamber receive a 17.4 ± 5.0-fold difference in UV-C dose, which yields an 8.2 ± 1.4-fold difference in SARS-CoV-2 log reduction. Vertical dashed lines indicate the normalized UV-C dose measured by either a PCI or radiometer at monitoring sites in the chamber corners and are colored according to the heatmap in (**A**). Black dashed line indicates linear least squares regression on data (y = mx + b; m = 3.114 (2.038, 4.19), b = 0.3342 (− 0.3062, 0.9766); numbers in parentheses represent a 95% confidence interval on the fit parameters). Pearson correlation coefficient r = 0.8376; p-value = 1.4428 × 10^–5^. (**C**) Normalized on-N95 SARS-CoV-2 dose–response curve for 2 N95 facepieces. Datapoints represent log_10_(normalized SARS-CoV-2 survival), where normalized SARS-CoV-2 survival is calculated as TCID_50_/mL divided by the time-matched negative control TCID_50_/mL. Black dashed line indicates linear regression on data (y = mx + b; m = − 0.0531 (− 0.06653, − 0.03961), b = − 0.2045 (− 0.6663, 0.2574). Pearson correlation coefficient r = − 0.8842; p-value = 1.0526 × 10^–7^. Based on linear regression, the estimated D_90_ dose is between 18.83 and 19.03 mJ/cm^2^, depending on whether the y-intercept value is ignored or considered, respectively. In both (**B**) and (**C**), blue dashed lines denote simultaneous functional 95% prediction interval for the linear least squares regression, which indicates where the true linear regression line lies, with 95% confidence.

UV-C dose and SARS-CoV-2 log reduction correspond well (Fig. 5A) and are positively and linearly correlated (Pearson correlation coefficient r = 0.8376; p = 1.4428 × 10^–5^. P-value corresponds to the null hypothesis that the slope of the linear regression is zero^44^; thus, a significant p-value indicates that UV-C dose and SARS-CoV-2 log reduction are significantly correlated) (Fig. 5B). SARS-CoV-2 dose response is still being investigated, but is expected to be primarily log-linear^41,45–47^ in agreement with other pathogens. While the dose response curve likely has shoulder and/or tailing behavior at the lower and upper ends^12^, these nonlinear regions may not be captured with the range and resolution of UV-C doses tested here. The dose required for 90% inactivation (D_90_) estimated from a linear regression on the dose–response curve (Pearson correlation coefficient r = − 0.8842) is ~ 19 mJ/cm^2^, higher than the D_90_ of ~ 1.4 mJ/cm^2^ for dried SARS-CoV-2 on a nonporous surface^45^, as expected (Fig. 5C). As discussed above, the UV-C dose yielding 3-log inactivation of SARS-CoV-2 from N95 coupons and facepieces in this study was > 20 × lower than the necessary doses reported in many previous studies^6,16–18,22^; we hypothesize the discrepancy may be due to the lack of soiling agent and high hydrophobicity of the N95s used in this study. To determine the absolute UV-C dose necessary for safe and effective implementation of N95 decontamination, pathogen dose–response should be carefully characterized with the specific N95 model and pathogen of interest using representative soiling conditions.

Similar to the coupon study, we observe varying SARS-CoV-2 inactivation among replicate inoculation sites receiving similar UV-C dose (1.1 ± 0.8-log difference in inactivation between replicates). We hypothesize that replicate-to-replicate variation in measured SARS-CoV-2 inactivation level in either the on-N95 and/or coupon studies may be due to: (1) the quantal nature of the TCID_50_ assay^48,49^, and/or (2) variability in the slope of the coupon surface caused by separation of N95 layers along the three sides without a seam (Supplementary Fig. S17). Slight variations in the amount of virus inoculated, viral extraction efficiency, and excision area may also contribute to technical variation in measured TCID_50_/mL. To characterize intra-chamber variation, we quantified the fold difference in UV-C dose and SARS-CoV-2 log reduction across both N95 facepieces in the chamber. Simulation predicted a 14.9-fold difference in UV-C dose across the facepieces of both N95s in the chamber, and we measured in situ a dose difference of 17.4 ± 5.0-fold. This UV-C dose range yielded an 8.2 ± 1.4-fold difference in SARS-CoV-2 log reduction (from 0.4 ± 0.1-log reduction at location f to 3.4 ± 0.4-log reduction at location a). The observed 2.9 ± 0.2-log difference in SARS-CoV-2 survival across N95s within one chamber is substantial, given that effective N95 decontamination has previously been defined in some studies as ≥3-log reduction of pathogens. To our knowledge, this is the first study to rigorously quantify both UV-C dose and viral inactivation at paired locations on intact N95s, to understand how UV-C dose distribution and resulting decontamination efficacy depend on N95 facepiece shape.

Because in-situ dose is often monitored at an off-N95 location in decontamination protocols^50^, we also investigated whether dose monitoring at low-irradiance locations on the chamber floor could directly indicate the lowest on-N95 UV-C dose (Note S4). The doses in the corners of the chamber floor were 49.5% ± 1.6% (radiometer location) and 44.0 ± 0.7% (floor PCI location) of the maximum on-N95 dose, whereas the lowest on-N95 dose measured was 6.0% ± 1.6% of the maximum on-N95 dose (Fig. 5A,B).Thus, in the UV-C chamber tested here, dose monitoring on the chamber floor cannot serve as a proxy for the lowest on-N95 UV-C dose.

### Intra-N95 variation in UV-C dose and SARS-CoV-2 inactivation

In addition to characterizing intra-chamber variation, we also analyzed UV-C dose and SARS-CoV-2 inactivation variation across each individual N95. On the front N95, the apex (location a) receives the highest dose while the more steeply sloped regions near the base of the sides of the N95 (location b) receive some of the lowest doses that can be measured on the front N95 with our approach, given the footprint of the PCI and SARS-CoV-2 inoculation site. Across the locations sampled on the front N95, simulation predicted a 3.0-fold difference in UV-C dose, and we measured a 2.8 ± 0.4-fold difference in UV-C dose using PCIs in situ. This variation in UV-C dose yielded a 2.8 ± 1.5-fold difference in SARS-CoV-2 log reduction (from 1.6 ± 1.2-log reduction at location b to 3.4 ± 0.4-log reduction at location a). While placing the N95 directly in the center of the UV-C chamber rather than offset toward the door would increase UV-C dose uniformity, throughput may be reduced, as the number of N95s that could fit in the chamber without contacting each other would be reduced from three to one.

Across the 3 measured locations on the facepiece of the corner N95, simulation predicted an 8.1-fold difference in UV-C dose and we measured a 10.2 ± 3.3-fold difference in dose. This variation in UV-C dose yielded a 4.9 ± 1.3-fold difference in SARS-CoV-2 log reduction (from 0.4 ± 0.1-log reduction at location f to 2.1 ± 0.7-log reduction at the maximum-dose location on the corner N95, which was either location d or e depending on the replicate). However, because the measurement locations were chosen to evenly sample the range of UV-C doses applied across both (front and corner) N95s, the measured locations on the corner N95 did not capture the maximum corner N95 dose near the apex (Figs. 4A, 5A). Thus, we expect that the total variation in UV-C dose and resulting viral inactivation on the corner N95 would be even higher than measured here.

We also compared the magnitude of variation in UV-C dose and SARS-CoV-2 inactivation on the front and corner N95s. As compared to the front N95, the corner N95 had greater intra-N95 variation in both UV-C dose and SARS-CoV-2 inactivation. In contrast to the front N95, which had an equal amount of variation (2.8-fold) in UV-C dose and SARS-CoV-2 log reduction, the difference in UV-C dose (10.2-fold) was greater than the difference in SARS-CoV-2 log reduction (4.9-fold) on the corner N95. We hypothesize that the corner N95 receives UV-C doses which may be in the shoulder of the SARS-CoV-2 survival curve, where SARS-CoV-2 inactivation is not fully log-linear with dose^12^. If the corner N95 receives UV-C doses in this shoulder region, the magnitude of intra-N95 UV-C dose variation will be larger than the amount of variation in SARS-CoV-2 inactivation.

Characterization of UV-C dose distribution across N95s within a decontamination system is valuable for informing decontamination protocols and evaluating throughput. In our system, we observed substantially lower variation in UV-C dose and SARS-CoV-2 inactivation across a single N95, as compared to across both N95s in the chamber, which suggests that approaches to increase decontamination throughput should be carefully considered. Including more N95s in the chamber may not necessarily increase throughput as compared to a single N95 in the center of the chamber, as multiple N95s likely have more nonuniform on-N95 dose because they are more spread out and can shadow each other. Greater UV-C dose nonuniformity increases the exposure time needed for all N95 surfaces to reach the minimally acceptable UV-C dose, which in turn affects the total number of safe reprocessing cycles prior to N95 material degradation (Fig. 1A)^25^. The simulation and in-situ dose measurement workflows we demonstrate here help inform N95 positioning within decontamination systems to optimize decontamination cycle time, pathogen inactivation, and the maximum number of safe reuses.

In summary, we have demonstrated that the N95 facepiece shape and position within a UV-C decontamination system have substantial influence on the on-N95 UV-C dose distribution and concomitant decontamination efficacy. We introduce a workflow to combine optical modeling and in-situ quantitative PCI dosimetry to characterize on-N95 UV-C dose with high spatial resolution, high throughput, and near-ideal angular response. For the first time, we combined simultaneous and robust quantitative UV-C dose measurements with SARS-CoV-2 inactivation measurements at specific locations on N95 respirators to probe the relationship between on-N95 dose and pathogen inactivation within each UV-C exposure. The substantial variation in on-N95 UV-C dose and SARS-CoV-2 inactivation we observed in a single decontamination chamber highlights how nonuniform UV-C dose distribution impacts pathogen inactivation and total UV-C exposure (which influences N95 material degradation and the safe number of decontamination cycles). We further demonstrated that a lower-cost colorimeter accurately quantifies dose from PCIs, making the PCI quantification workflow more accessible. Additional investigation into alternative color metrics^29^ may extend the dynamic range of PCIs measured with lower-cost color readers.

The approaches introduced here for characterizing UV-C dose and pathogen inactivation with cup-shaped 3M 1860 N95s are versatile and widely applicable to other N95 models or other substrates. The location and spatial resolution of in-situ UV-C dose measurements are limited only by PCI size and readout resolution. In this study, PCI size was limited by the spectrocolorimeter aperture size; however, alternative color readout methods^29^ using a camera, flatbed scanner, or other imaging modalities can analyze smaller PCI regions for finer in-situ dose measurement resolution. Additionally, PCI flexibility facilitates measurement on a variety of nonplanar N95 surfaces, though measurements of irregular topologies to which one large PCI cannot conform may be infeasible or may require an impractical number of measurements with small PCIs on each individual face. Optical simulations are also applicable to any N95 shape that can be accurately captured using a 3D scanner. However, for N95 models with greater N95-to-N95 variability in shape, small differences in N95 shape (between simulation and in-situ experiments, or from run-to-run) may substantially impact the resulting UV-C dose distribution. For example, small changes in the pleat configuration and/or opening angle of duckbill and pleated N95s may yield large differences in dose distribution due to shadowing and angle-dependent UV-C dose. The rapid iterations possible with optical simulation can provide preliminary evaluations of UV-C dose distribution sensitivity to N95 shape variability, highlighting cases in which in-situ dose measurements with PCIs may be particularly valuable. Likewise, pathogen inactivation can be measured on a variety of N95 models^16,22^, as long as the pathogen sample can be accurately applied and dried on the N95 location of interest (some hydrophobic and/or steeply sloped N95 regions may be difficult to inoculate without sample migration from the region of interest).

Future studies are needed to characterize SARS-CoV-2 dose response in more clinically relevant conditions, such as with the addition of soiling agents and on varying N95 models. Extending the dynamic range of PCIs, while maintaining a near-ideal angular response, is also critical for measurement of ⪆260 mJ/cm^2^ UV-C dose on-N95. Overall, the on-N95 UV-C dosimetry approach here facilitates characterization of decontamination protocols of any UV-C system, supporting system-specific validation that is critical to ensuring safe and effective N95 decontamination.

## Supplementary Information

Supplementary Information.

## Data Availability

Additional data from the current study are available from the corresponding author upon reasonable request.
